# Involvement of Toll Like Receptor 2 Signaling in Secondary Injury during Experimental Diffuse Axonal Injury in Rats

**DOI:** 10.1155/2017/1570917

**Published:** 2017-02-15

**Authors:** Yonglin Zhao, Junjie Zhao, Ming Zhang, Yahui Zhao, Jiaxi Li, Xudong Ma, Tingqin Huang, Honggang Pang, Bo Wang, Jinning Song

**Affiliations:** ^1^Department of Neurosurgery, The First Affiliated Hospital of Xi'an Jiaotong University, 277 West Yanta Road, Xi'an, Shaanxi 710061, China; ^2^Department of Neurosurgery, The Second Affiliated Hospital of Xi'an Jiaotong University, No. 157 Xiwu Road, Xi'an, Shaanxi 710004, China

## Abstract

Treatment of diffuse axonal injury (DAI) remains challenging in clinical practice due to the unclear pathophysiological mechanism. Uncontrolled, excessive inflammation is one of the most recognized mechanisms that contribute to the secondary injury after DAI. Toll like receptor 2 (TLR2) is highlighted for the initiation of a vicious self-propagating inflammatory circle. However, the role and detailed mechanism of TLR2 in secondary injury is yet mostly unknown. In this study, we demonstrated the expression of TLR2 levels in cortex, corpus callosum, and internal capsule and the localization of TLR2 in neurons and glial cells in rat DAI models. Intracerebral knockdown of TLR2 significantly downregulated TLR2 expression, attenuated cortical apoptosis, lessened glial response, and reduced the secondary axonal and neuronal injury in the cortex by inhibiting phosphorylation of mitogen-activated protein kinases (MAPK) including Erk, JNK, and p38, translocation of NF-*κ*B p65 from the cytoplasm to the nucleus, and decreasing levels of proinflammatory cytokines including interleukin-6, interleukin-1*β*, and tumor necrosis factor-*α*. On the contrary, administration of TLR2 agonist to DAI rats achieved an opposite effect. Collectively, we demonstrated that TLR2 was involved in mediating secondary injury after DAI by inducing inflammation via the MAPK and NF-*κ*B pathways.

## 1. Introduction

Diffuse axonal injury (DAI), an important aspect of traumatic brain injury (TBI), is getting increasing recognition as a major cause of long-term disability and mortality [1]. As the pathogenesis of DAI is unclear, there is no effective treatment for axonal disconnection. Secondary injury after DAI results from a series of biochemical events including metabolism, electrochemistry, and inflammation [2]. Recently, attention has been raised to the inflammation activated by injured tissue. Activation of innate immune response initiates a cascade of inflammatory signaling pathways and subsequent biochemical responses [3], which eventually leads to a variety of cellular disturbances such as microtubules dissolution, axoplasmic perturbation, glial response, and even cell death.

Pattern-recognition receptors (PRRs), such as membrane-bound toll-like receptors (TLRs), primarily expressed in microglia and astrocytes in the central nervous system (CNS), can recognize endogenous danger-associated molecular patterns (DAMPs) released by dying or damaged cells when cellular stress takes place [4]. A specific member of the TLR family, namely, TLR2, functions as a master sentry receptor to detect neuronal cell death and tissue damage in many different neurological conditions. In TLR2 signaling pathway, some adaptor molecules are involved in modulating the activation of nuclear factor-*κ*B (NF-*κ*B) and the mitogen-activated protein kinase (MAPKs), resulting in the induction of genes involved in inflammatory responses, such as the release of proinflammatory cytokines, including IL-1*β* (interleukin-1*β*), IL-6 (interleukin-6), and TNF-*α* (tumor necrosis factor-*α*) [5–8]. Several studies have confirmed that TLR2 signaling plays a critical role in axonal injury. TLR2 orchestrated the innate immune response leading to efficient and rapid clearance of myelin debris and nerve regeneration after sciatic nerve lesion [9, 10]. TLR2 agonist robustly increased the microglial response to ablation and reduced secondary degeneration of central myelinated fibres after spinal cord injury [11]. However, the role of TLR2 in the secondary injury following DAI remains mostly unknown.

This study was conducted to determine the role of TLR2 in the development of secondary injury following DAI and to try to investigate the potential mechanism involved. In this study, we observed temporal expression and localization of TLR2 after DAI. Our results showed that down- and upregulation of TLR2 modulated axonal pathological changes, glial response, cell death, and release of inflammatory cytokines following DAI through the MAPK and NF-*κ*B pathways [12–14].

## 2. Methods

### 2.1. Animals and Grouping

All experiments were performed on male adult Sprague–Dawley (SD) rats (weighing 250–300 g, 8–10 weeks old) provided by the Experimental Animal Center of Xi'an Jiaotong University (License no. SCXK (Shaanxi) 2006-001). Rats were housed in groups of four per cage, maintained at an ambient temperature of 22 ± 1°C, on a 12-h light/dark cycle, with ad libitum access to food/water. All protocols and procedures were approved by the Biomedical Ethics Committee of Animal Experiments of Shaanxi Province in China and complied with the principles and procedures of the Guidance Suggestions for the Care and Use of Laboratory Animals formulated by the Ministry of Science and Technology of PRC. Every effort was made to minimize the discomfort of the rats during surgery or recovery from surgery.

In the experiment, each test was performed independently for three times per rat (6 or 12 rats per group). 102 rats were used in total. SD rats were divided into the following groups.

To investigate the temporal expression of TLR2, rats were assigned to control group (12 rats), DAI 1 d group (6 rats), DAI 3 d group (12 rats), and DAI 7 d group (6 rats), according to different time points after DAI.

To examine whether TLR2 expression could be reduced in rat brain by siRNA, rats were assigned to control siRNA group (6 rats, control siRNA was intracerebroventricularly (ICV) injected to normal rats and brains were harvested at 3 d after injection), 1 d posttransfection group (6 rats, TLR2 siRNA was ICV injected to normal rats and brains were harvested at 1 d after injection), 3 d posttransfection group (6 rats, same as 1 d posttransfection group, but brains were harvested at 3 d after injection), DAI 3 d + TLR2 siRNA group (12 rats, TLR2 siRNA was ICV injected within 20 min after DAI and brains were harvested at 3 d after DAI), and DAI 3 d + control siRNA group (6 rats, same as DAI 3 d + TLR2 siRNA group, but rats were treated with control siRNA).

Pam3CSK4 (InvivoGen, San Diego, CA, USA) was dissolved in sterile endotoxin-free water (vehicle) with the final concentration of 1 mg/ml and injected intravenously by cauda vein for 3 successive days after DAI. To determine the optimum concentrations of Pam3CSK4, rats were assigned to the following groups: DAI 3 d + vehicle group (6 rats); DAI 3 d + Pam3CSK4 (0.5 mg/kg) group (6 rats); DAI 3 d + Pam3CSK4 (1 mg/kg) group (12 rats); DAI 3 d + Pam3CSK4 (2 mg/kg) group (6 rats).

### 2.2. Animal Model of DAI

DAI model was established using a lateral head rotation device according to our previous study [15]. After anesthesia with intraperitoneal injection of 1% (w/v) pentobarbital sodium (35 mg/kg), the rat head was horizontally secured to the device by a head clip anchoring two lateral ear bars and an anterior teeth hole, with its body at 30° oblique to the top of the laboratory table. When the trigger was pushed, the rat head was rapidly rotated by 90°, undergoing a sudden acceleration and deceleration. Control rats only underwent anesthesia and fixation to the device without subjection to injury.

DAI rats were rendered comatose due to injury and regained consciousness after around half an hour. During this period, rats were placed in open and ventilated environment and the changes of vital signs of rats were observed to ensure that rats would not get suffocated. Moreover, rats exhibited abnormal behaviors for approximately 12 hours after coma, with a weakened response to stimulation, an unstable gait, and reduced activity and food intake. Rats in the control group behaved normally after anesthesia, with normal activity and rapid responses. Rats were still maintained on free access to food/water, with a 12 h light-dark cycle at a temperature of 22 ± 2°C until the time of euthanasia. For morphology staining, rats were perfused with 200 ml normal saline followed by 200 ml 4% (w/v) buffered paraformaldehyde, pH 7.4 after euthanasia with an intraperitoneal injection of 5% (w/v) pentobarbital sodium (150 mg/kg). The brains were removed, postfixed, embedded in paraffin, and cut into sections (5 *μ*m) with a rotary microtome. For western blot and ELISA, rats were only perfused with 200 ml normal saline. After the brains were removed, cortex tissues were separated and preserved in liquid nitrogen. Four rats that died throughout the experiment were excluded and later replaced by new rats.

### 2.3. In Vivo TLR2 siRNA Transfer

We performed in vivo transfer according to the method described in previous study [16]. SiRNA duplexes targeting the coding region of rat TLR2 were purchased from ON-Target siRNA (ThermoFisher Scientific, Pittsburgh, PA, USA). Scrambled control siRNA was similarly produced from a scrambled sequence. TLR2 siRNA or control siRNA was diluted with the same volume of in vivo transfection reagent (v/v) (Entranster™-in vivo; Engreen, Beijing, China) [17]. The solution was mixed gently by pipetting up and down for 15 minutes at room temperature. 10 *μ*L (1 mM) transfection reagent-in vivo–siRNA mixture was ICV injected using a Hamilton microsyringe under a guidance of stereotaxic instrument (Kent Scientific Co., Torrington, CT, USA) within 20 min. After each rat was anesthetized, the scalp was incised, and the bregma was exposed. Then, craniotomy was performed, diameter less than 1.5 mm. The stereotaxic coordinates were 0.8 mm posterior, 1.5 mm lateral to the bregma, and 4.5 mm ventral to the surface of the skull. The needle was held in place for 10 minutes after injection [18, 19]. The craniotomy was then sealed with bone wax, and the scalp was closed with sutures. Body temperature was maintained at 37°C throughout the procedure. Rats in other groups had the same operation except ICV injection.

### 2.4. RNA Extraction and Real-Time Quantitative PCR (Q-PCR)

Total RNA was extracted from rat cortex using TRIzol Reagent (ThermoFisher Scientific, Pittsburgh, PA, USA) according to the manufacturer's instructions. The primers used for real-time quantitative PCR are as follows: TLR2 (93 bp), 5′-TATCAGTCCCAAAGTCTAAAGTCG-3′ (forward) and 5′-CTACCTCCGACAGTTCCAAGATG-3′ (reverse) [20]. The reaction conditions were as follows: 95°C for 30 seconds, 40 cycles of 95°C for 5 seconds, and 60°C for 30 seconds. We used the cycle threshold (CT) as the representative point. The relative expression of TLR2 mRNA in each group (fold-change compared with control) was calculated using the formula: RQ = 2^−ΔΔCt^. Each reaction was performed in triplicate.

### 2.5. Transmission Electron Microscopy (TEM)

Samples for transmission electron microscopy were prepared as previously described [21]. Areas of interest were cut from the cortex, trimmed into approximately 1 mm^3^ blocks, and then postfixed at 4°C until processing. Samples were washed in 0.1 mol/L phosphate buffer saline (PBS) for 30 min, fixed in 1% (w/v) osmium tetroxide for 2 h at 4°C, and then washed in PBS for 10 min. Subsequently, sample blocks were dehydrated with an ethanol series, embedded in Epon 812 resin, and then cut into semithin sections (1-2 *μ*m). Following methylene blue staining, semithin sections were cut into thin sections (50–70 nm) using an ultrathin microtome. Finally, thin sections were lightly counterstained with 2% (w/v) uranyl acetate and 3% (w/v) lead citrate prior to examination with TEM (H-7650, Hitachi, Tokyo, Japan).

### 2.6. Hematoxylin and Eosin (H&E) Staining and Golgi Silver Staining

The sections were stained with hematoxylin and eosin and processed for Golgi staining according to the manufacturer's instructions for the FD Rapid GolgiStain™ Kit (FD NeuroTechnologies, Inc., Columbia, MD, USA), respectively. All the sections were observed with a light microscope (BX-40, Olympus, Tokyo, Japan) at 40x magnification.

### 2.7. Immumohistochemical Staining

The sections were deparaffinized, rehydrated, and incubated with 3% (v/v) H_2_O_2_ to block the endogenous peroxidase activity. Antigen unmasking was performed using high temperature epitope retrieval. Sections were then blocked in 2% (v/v) normal donkey serum and incubated in primary antibodies including rabbit polyclonal TLR2 (1 : 100, Bioss, Beijing, China), rabbit monoclonal *β*-APP (1 : 200, Abcam, Cambridge, UK), mouse monoclonal tau46 (1 : 300, Cell Signaling Technology, Danvers, MA, USA), rabbit monoclonal neurofilament-L (NF-L, 1 : 100, Cell Signaling Technology, Danvers, MA, USA), mouse monoclonal GFAP (1 : 400, Cell Signaling Technology, Danvers, MA, USA), rabbit monoclonal vimentin (1 : 400, Cell Signaling Technology, Danvers, MA, USA), and rabbit monoclonal Iba-1 (1 : 400, Wako, Osaka, Japan) overnight at 4°C. Sections were washed on the following day and incubated for 1 h in donkey anti-mouse, anti-rabbit, or anti-goat HRP-conjugated secondary antibodies (1 : 500). Visualization of the antigen of interest was achieved after 5 min of exposure to diaminobenzidine. Results were observed under a 40x light microscope. The region selected to observe was located in frontal cortex and corpus callosum from brain midline to lateral ventricle at bregma level −3, as well as infernal capsule at the same level. The integral optical density (IOD) of positive cells in 10 randomly selected areas was measured with an image analysis program (Image-Pro Plus 6.0, Media Cybernetics, Bethesda, MD, USA) [22].

### 2.8. Immunofluorescence Staining

The brain-tissue processing methods were the same as described above (immunohistochemical staining). Briefly, the sections were deparaffinized, rehydrated, and blocked using 3% (v/v) H_2_O_2_. Antigen unmasking was performed using high temperature epitope retrieval. Sections were then blocked in 2% (v/v) normal donkey serum and incubated in primary antibodies including rabbit antibody against rabbit polyclonal TLR2, mouse monoclonal NeuN (1 : 400, Millipore, Billerica, MA, USA), and mouse monoclonal GFAP and goat polyclonal Iba-1 (1 : 200, Abcam, Cambridge, UK). The rat brain sections were incubated overnight at 4°C temperature. Alexa 488 and Alexa 568 secondary fluorescent antibodies (1 : 400, Invitrogen, Carlsbad, CA, USA) were used for one hour at 37°C, and the nuclei were stained with 4′,6-diamidino-2-phenylindole (DAPI, 1 *μ*g/ml) for ten minutes. The sections were observed using a fluorescence microscope (Molecular Devices, Sunnyvale, CA, USA).

### 2.9. Terminal Deoxynucleotidyl Transferase-Mediated Digoxigenin-DUTP-Biotin Nick-End Labeling (TUNEL) Assay

The DeadEnd™ Fluorometric TUNEL System (Promega, Madison, Wisconsin, USA) was used to detect apoptosis in rat cortex. The TUNEL assay was performed following manufacturer's instructions. Briefly, the sections were deparaffinized, rehydrated, and immersed in 0.2% (v/v) Triton® X-100 in PBS for 5 minutes. Sections were incubated with equilibration buffer and equilibrated at room temperature for 10 minutes, followed by TdT reaction incubation for 60 minutes at 37°C. The reaction was stopped and sections were washed with PBS. The nuclei were stained with DAPI for ten minutes. Green fluorescence of apoptotic cells and blue fluorescence of nuclei were detected by fluorescence microscopy. 10 nonadjacent fields in frontal cortex were randomly chosen to count the number of TUNEL positive cells. The total counts were converted to an apoptotic index (AI), which was defined as the number of TUNEL positive cells per mm^2^. AI = number of apoptosis cells/(10 × 0.5024).

### 2.10. Western Blot

Total protein from cortex tissue was purified using RIPA. The protein concentrations of extracts were determined using a bicinchoninic acid (BCA) protein assay reagent kit. Protein samples (40 *μ*g) were analyzed by electrophoresis on 10% SDS-PAGE gels. Equal amounts of proteins per lane were electrophoretically transferred onto immobilon-P/PVDF membranes (Millipore Corp, Billerica, MA, USA). Then membranes were blocked with 5% (w/v) skim milk powder in TBST buffer (0.05 M Tris pH 7.4, 0.15 M NaCl, 0.05% Tween 20) and incubated overnight at 4°C with rabbit polyclonal TLR2 antibody (1 : 500), rabbit monoclonal *β*-actin antibody (1 : 1000, Cell Signaling Technology, Danvers, MA, USA), mouse monoclonal tau46 (1 : 1000), rabbit monoclonal *β*-APP (1 : 2000), rabbit monoclonal NF-*κ*B (1 : 1000, Cell Signaling Technology, Danvers, MA, USA), rabbit monoclonal phospho-NF-*κ*B (Ser536, 1 : 1000, Cell Signaling Technology, Danvers, MA, USA), rabbit polyclonal p38 MAPK (1 : 1000, Cell Signaling Technology, Danvers, MA, USA), rabbit polyclonal phospho-p38 MAPK (Thr180/Tyr182, 1 : 1000, Cell Signaling Technology, Danvers, MA, USA), rabbit polyclonal phospho-p44/42 MAPK (extracellular signal-regulated kinases 1/2, Erk1/2) (Thr202/Tyr204, 1 : 1000, Cell Signaling Technology, Danvers, MA, USA), rabbit polyclonal p44/42 MAPK (1 : 1000, Cell Signaling Technology, Danvers, MA, USA), rabbit polyclonal phospho-JNK (c-Jun N-terminal kinases 1/2) (Thr183/Tyr185, 1 : 1000, Cell Signaling Technology, Danvers, MA, USA), and rabbit polyclonal JNK (1 : 1000, Cell Signaling Technology, Danvers, MA, USA). After incubation, membranes were washed three times with TBST and then incubated with goat anti-rabbit or anti-mouse IgG-HRP secondary antibody (1 : 5000, Abcam, Cambridge, UK) for 1 h at room temperature with subsequent washing in TBST. The membranes were visualized using ChemiDoc MP System (Bio-Rad protein assay, Bio-Rad, Segrate, Italy) with ECL substrate (Millipore, Billerica, MA, USA). *β*-Actin or lamin B was chosen as an internal control to ensure equivalent amounts of protein. Densitometric quantification of the bands was performed using Image J software (version 1.29x: NIH, Bethesda, MD, USA).

### 2.11. Enzyme-Linked Immunosorbent Assay (ELISA) Analysis

Cortex tissue was collected and homogenized with ethylene-diamine tetra-acetic acid-free protease inhibitor cocktail tablets using 50 *μ*L/10 mg of tissue. The homogenates were centrifuged at 4°C at 12000 ×g for 15 minutes and supernatants were collected carefully. Total protein content for each sample was determined using BCA protein assay reagent kit. Protein samples (50 *μ*g) were evaluated in duplicate using IL-1*β*, IL-6, and TNF-*α* assay kits (R&D Systems, Minneapolis, MN, USA), in accordance with the manufacturer's guidelines. Tissue cytokine concentrations were expressed as picograms per milligram of protein.

### 2.12. Statistics Analysis

SPSS 18.0 (SPSS, Chicago, IL, USA) was used for statistical analyses. All data were presented as mean ± SD. We used one-way ANOVA to compare numerical data in more than 2 groups, followed by LSD (L) to conduct a post hoc test. A *p* value less than 0.05 was considered statistically significant.

## 3. Results

### 3.1. Pathological Changes in DAI Model Rats

**Figure 1:**
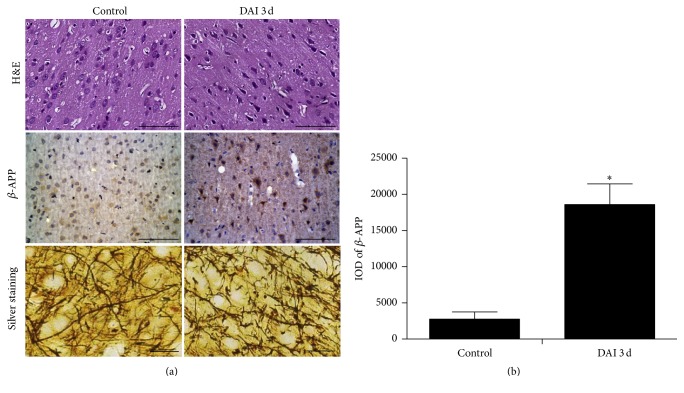
Pathological changes after DAI in rat cortex. (a) Pathological changes in rat cortex of DAI 3 d and control groups were confirmed by H&E staining, *β*-APP immunohistochemistry (scale bar = 100 *μ*m), and silver stain (scale bar = 20 *μ*m). (b) The bar graphs showed the statistical results for *β*-APP expression in the cortex (*n* = 6; ^*∗*^*p* < 0.05, compared with control group). The relative increase in *β*-APP levels was calculated in 10 random fields (40x magnification) and assessed by densitometry (IOD). Data were presented as mean ± SD in three separate experiments.

In order to evaluate the validity of DAI model, histopathological features in the injured cortex were examined using H&E staining, *β*-APP immunohistochemistry, and silver-staining. On H&E-stained sections, neuronal pyknosis, swelling, torsion, and cell body deformation were present in rat cortex of DAI 3 d group while, in the control group, no similar abnormal histopathological changes were observed (Figure 1). Diffuse *β*-APP positive neurons in the cortex were observed and markedly increased in DAI 3 d group comparing to control group (Figure 1). Silver-stained sections revealed smooth, continuous, regularly shaped axons in the cerebral cortex of control group, while, in DAI 3 d group, a subset of axons exhibited bead-like structures and axonal retraction balls (Figure 1).

### 3.2. Temporal Expression and Localization of TLR2 after DAI

**Figure 2:**
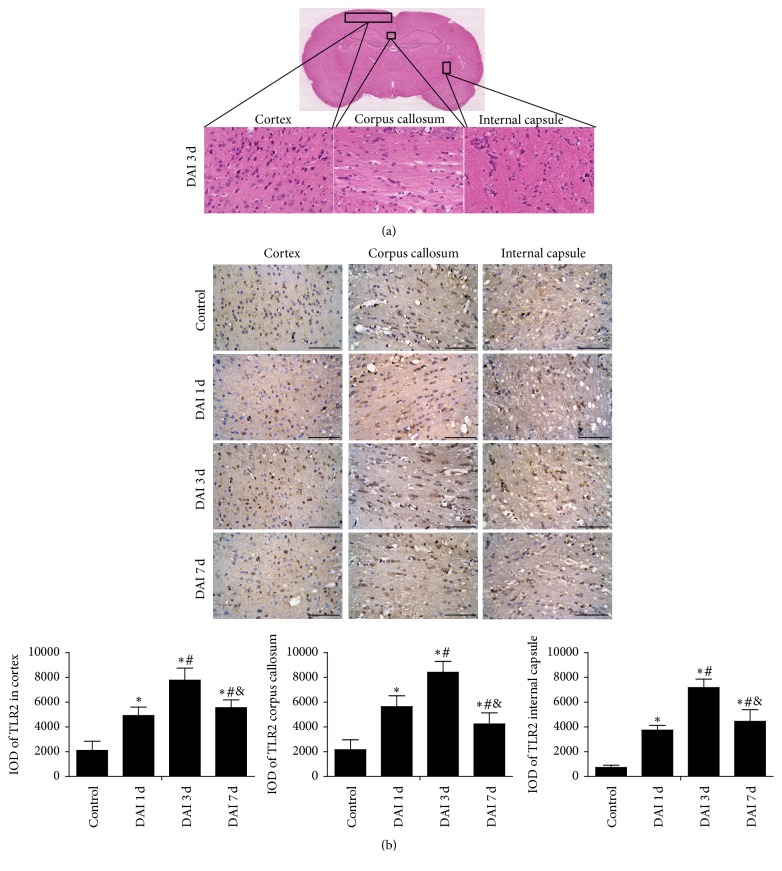
Temporal expression of TLR2 in rat brain after DAI. (a) A coronal brain map showed the region where the TLR2 expression was observed in frontal cortex, corpus callosum (from brain midline to lateral ventricle, at bregma level −3), and internal capsule. (b) The expression of TLR2 in rat cortex, corpus callosum, and internal capsule was measured at 1 d, 3 d, and 7 d after DAI by immumohistochemical staining (scale bar = 100 *μ*m; *n* = 6). The bar graphs showed the statistical results for TLR2 expression (^*∗*^*p* < 0.05, compared with control group; ^#^*p* < 0.05, compared with DAI 1 d group; ^&^*p* < 0.05, compared with DAI 3 d group). TLR2 positive cell levels were counted in 10 random fields (40x magnification). The relative increase in TLR2 level was assessed by densitometry (IOD). Data was presented as mean ± SD in three separate experiments.

**Figure 3:**
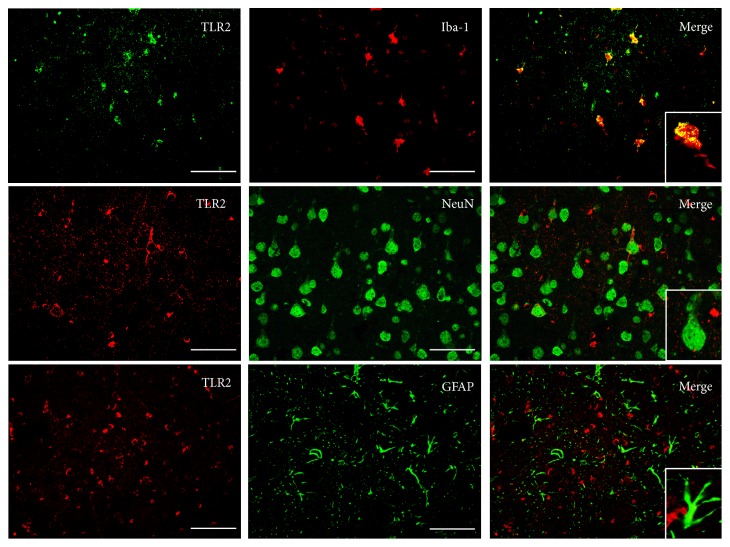
Localization of TLR2 in rat cortex at 3 d after DAI. Double immunofluorescent staining was performed with antibody to TLR2 (red or green) and antibodies to NeuN (marker for neurons, green), GFAP (marker for astrocytes, green), and Iba-1 (marker for microglia, red), respectively (scale bar = 50 *μ*m; *n* = 6). The small figures located in the bottom right corner were the magnification of particular sections.

The expression of TLR2 in rat cortex, corpus callosum, and internal capsule was measured at 1 d, 3 d, and 7 d after DAI. The levels of TLR2 in these areas all increased at 1 d, reached a peak at 3 d, and sustained until 7 d compared to control group following DAI (Figure 2). To further elucidate the distribution of TLR2 in rat cortex at DAI 3 d, we costained for neuron-specific (NeuN) and astrocyte-specific (GFAP) and for microglia specific (Iba-1) markers. The results showed that TLR2 levels were predominantly expressed in microglia after DAI (Figure 3).

### 3.3. TLR2 Expression Was Reduced in Rat Brain by siRNA

**Figure 4:**
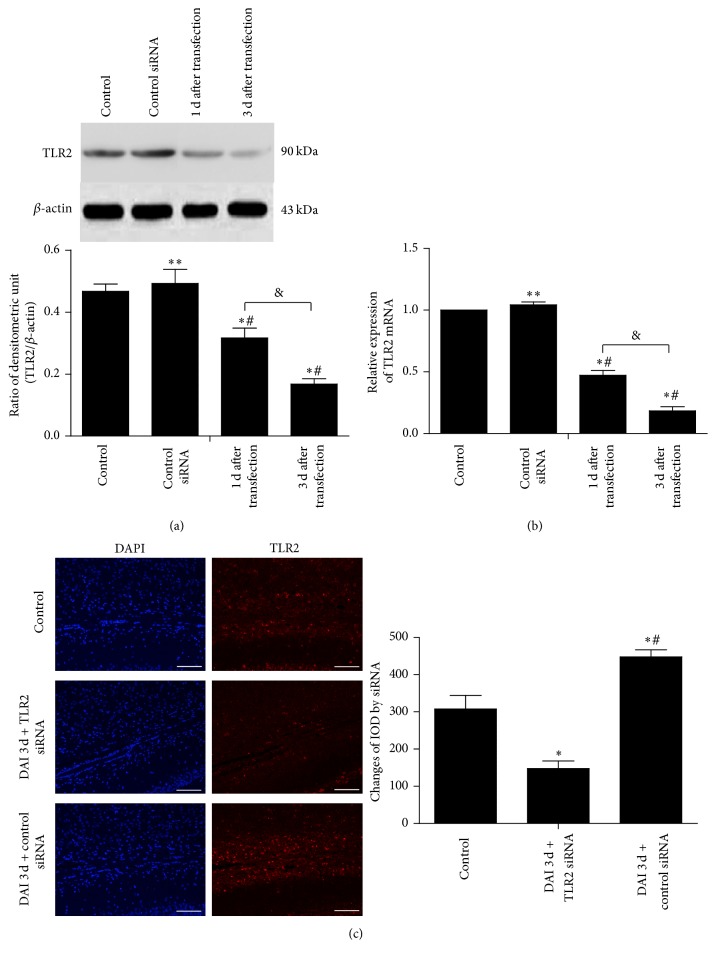
Intracerebroventricular injection of TLR2 siRNA attenuated TLR2 expression. The feasibility and efficiency of TLR2 siRNA transfection were assessed. ((a), (b)) The normal brain was treated with TLR2 siRNA or control siRNA and cortexes were collected 1 d or 3 d later and subjected to western blot analysis and RT-PCR to measure TLR2 level, respectively. The expression of *β*-actin was used as an internal control. Values were presented as mean ± SD (*n* = 6; ^*∗∗*^*p* > 0.05, ^*∗*^*p* < 0.05, compared with the control group; ^#^*p* < 0.05, compared with the control siRNA group; ^&^*p* < 0.05, compared with the 1 d postinfection group). (c) To confirm that TLR2 siRNA was effectively maintained in the DAI rats, immunofluorescence staining was carried out using antibodies to TLR2 (red) and DAPI (blue) in corpus callosum, respectively (scale bar = 100 *μ*m). The relative changes in TLR2 level were assessed by integral optical density (IOD). Values were presented as mean ± SD in three separate experiments (*n* = 6; ^*∗*^*p* < 0.05, compared with the control group; ^#^*p* < 0.05, compared with the DAI 3 d + TLR2 siRNA group).

To determine the feasibility and efficiency of siRNA transfection, the normal rats were treated with TLR2 siRNA by intracerebroventricular injection and accessed by western blot and RT-PCR. The results showed that intracerebroventricular injection of TLR2 siRNA could reduce the expression of TLR2 mRNA and levels of TLR2 protein in normal brain at 1 d and 3 d after injection. Compared to 1 d postinjection group, the expression of TLR2 mRNA and levels of TLR2 protein in 3 d postinjection group were much lower (Figures 4(a) and 4(b)). To confirm that TLR2 siRNA still effectively functioned in the DAI rats, TLR2 immunostaining was performed to assess the expression of TLR2 protein in DAI rats after treatment with the control or TLR2 siRNA. TLR2 was at a relatively low level in control group and was significantly upregulated in DAI 3 d + control siRNA group in the corpus callosum, while a significant reduction of TLR2 expression was observed in DAI 3 d + TLR2 siRNA group compared with control and DAI 3 d + control siRNA groups (Figure 4(c)).

### 3.4. Identification of Optimal Concentration of Pam3CSK4

**Figure 5:**
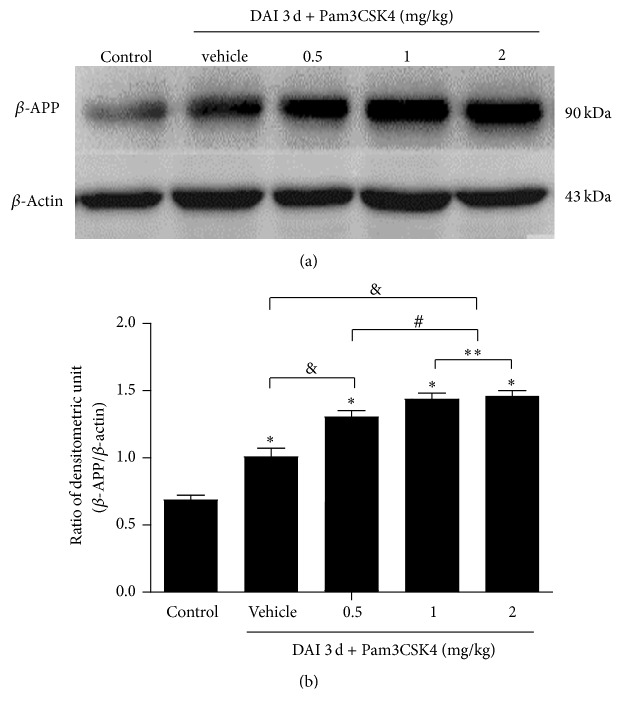
Identification of optimal concentration of Pam3CSK4. (a) The optimal doses of Pam3CSK4 (0.5, 1, or 2 mg/kg) were determined by western blotting of *β*-APP. *β*-Actin was used as an internal control. (b) The bar graphs showed the statistical results for *β*-APP expression. Values were presented as mean ± SD (*n* = 6; ^*∗*^*p* < 0.05, compared with control group; ^&^*p* < 0.05, compared with DAI 3 d + vehicle group; ^#^*p* < 0.05, compared with DAI 3 d + Pam3CSK4 (0.5 mg/kg); ^*∗∗*^*p* > 0.05, compared with DAI 3 d + Pam3CSK4 (1 mg/kg)).

To confirm the optimal dose of Pam3CSK4 impacting the secondary injury, Pam3CSK4 (InvivoGen, San Diego, CA, USA) was intravenously injected to the caudal vein within five minutes, respectively, at dose of 0.5, 1, and 2 mg/kg, once daily for 3 successive days after DAI according to previous studies [13, 23]. DAI was assessed by *β*-APP. Results showed that 1 mg/kg or 2 mg/kg Pam3CSK4 significantly increased *β*-APP expression, but *β*-APP expression did not significantly differ between these two groups (Figure 5). Accordingly, in our following research, we chose 1 mg/kg as the administration dose of Pam3CSK4 at a frequency of once daily for 3 successive days.

### 3.5. Role of TLR2 on Neuronal and Axonal Damage after DAI

**Figure 6:**
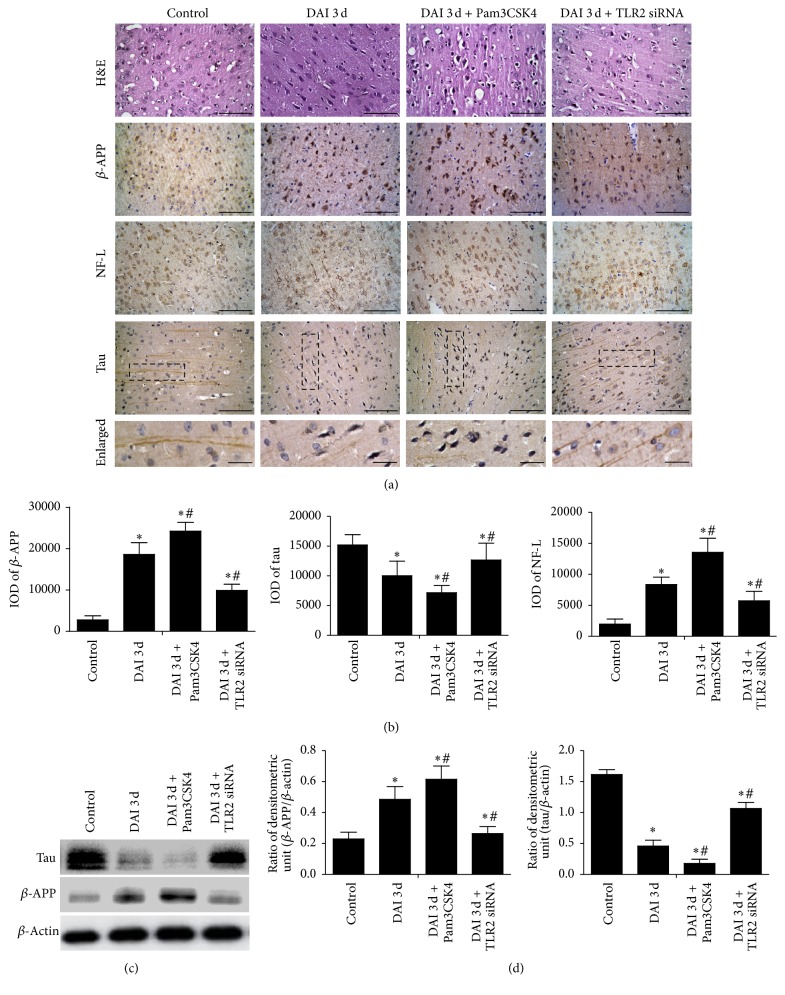
Role of TLR2 on secondary neuronal and axonal injury after DAI. (a) To confirm the role of TLR2 on the secondary neuronal injury, immumohistochemical staining of *β*-APP, tau, NF-L, and H&E staining in the rat cortex was performed (scale bar = 100 *μ*m). (b) The bar graphs showed the statistical results for *β*-APP, tau, and NF-L assessed by immunohistochemistry. The relative changes in *β*-APP, tau, and NF-L levels were counted in 10 random fields of relevant region (40x magnification) and assessed by densitometry (IOD). (c) The expression of *β*-APP and tau in each group was determined by western blotting. *β*-Actin was used as an internal control. (d) The bar graphs showed the statistical results for *β*-APP and tau expression. Data were presented as mean ± SD in three separate experiments (*n* = 6; ^*∗*^*p* < 0.05, compared with control group; ^#^*p* < 0.05, compared with DAI 3 d group).

H&E staining showed that, comparing to DAI 3 d group, neuronal pyknosis, swelling, torsion, cell body deformation, and expanded extracellular space of cell were more aggravated in the DAI 3 d + Pam3CSK4 group, while the similar pathological changes were rather mitigatory in the DAI 3 d + TLR2 siRNA group. The number of diffuse *β*-APP and NF-L positive neurons and staining intensity in the DAI 3 d + Pam3CSK4 group were markedly increased, while the expression of *β*-APP and NF-L significantly decreased in the DAI 3 d + TLR2 siRNA group, compared to DAI 3 d group. The tau immunohistochemical staining showed that, in control group, the axons were tau positive and regions of undulating distortions were fewer. In DAI 3 d group, a portion of axons, in which tau was nearly negative-stained, were found to display multiple regions of undulating distortions along their length. In the DAI 3 d + Pam3CSK4 group, lots of axonal tau were nearly negative-stained and axon morphology could not be observed, while in the DAI 3 d + TLR2 siRNA group, axon morphology was nearly normal and the axons were tau positive (Figure 6).

**Figure 7:**
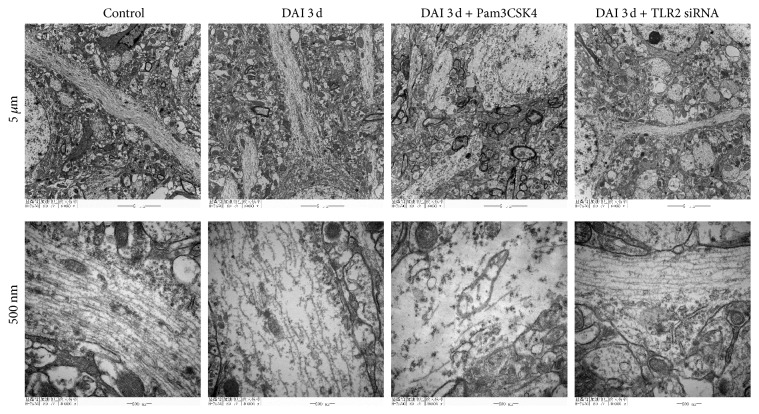
Role of TLR2 in ultrastructural alterations of axonal after DAI. Ultrastructural alterations of the longitudinal sections of axons after DAI were observed by TEM examination (scale bar = 5 *μ*m and 500 nm, resp.; *n* = 6).

TEM examination of the longitudinal sections of axons demonstrated ultrastructural alterations of the axonal microtubule lattice induced by DAI (Figure 7). The microtubules were identified as dark filamentous structures that traversed the main axis of an axon. In the control group, the normal microtubules were consecutive, integrated, and compact. In the DAI 3 d group, the arrangement of microtubules was disordered and the continuity of microtubules was lost. The microtubules were frayed and displayed conspicuous free ends. In the DAI 3 d + Pam3CSK4 group, the structure of microtubules collapsed and turned absent, and the axons were swollen. In the DAI 3 d + TLR2 siRNA group, a portion of microtubules were reserved and remained intact, but the number, integrity, and compactness of microtubules were still less than normal microtubules.

### 3.6. TLR2 Expression Was Related to Cell Apoptosis after DAI

**Figure 8:**
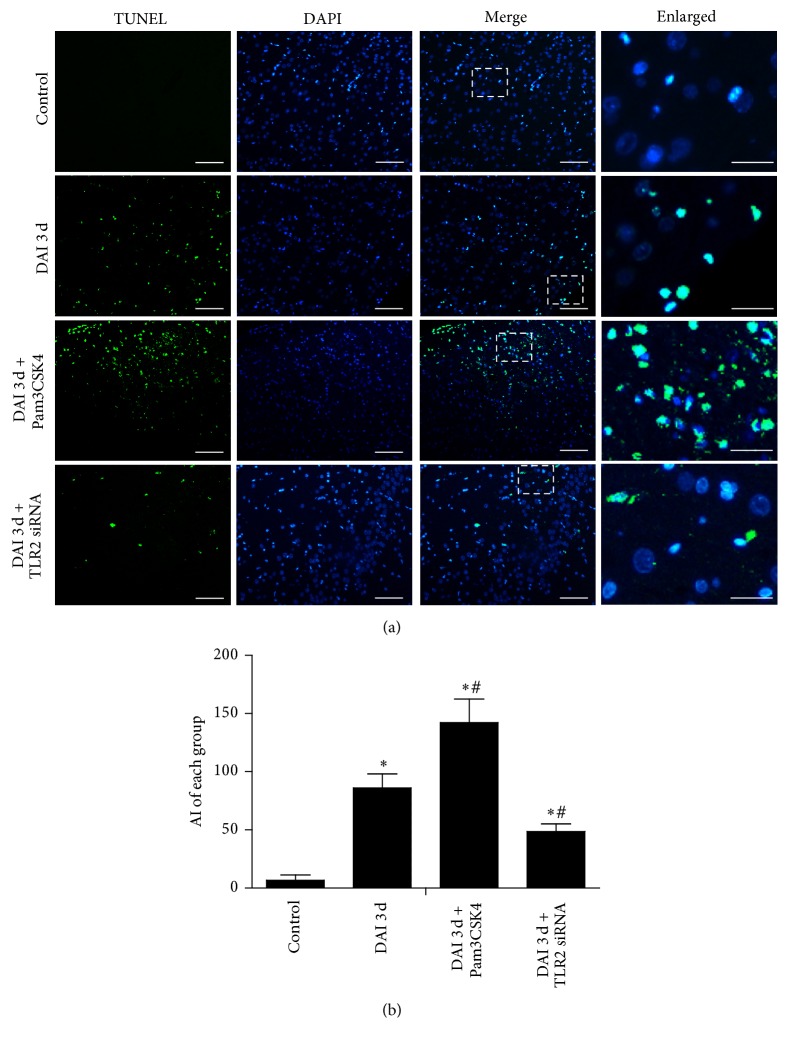
TLR2 signaling was related to cell apoptosis after DAI. TUNEL assay was used to detect apoptotic cells in rat cortex. (a) Colocalization of TUNEL (green) and nuclei (DAPI, blue) were considered as apoptotic cells (scale bar = 100 *μ*m; enlarged figures scale bar = 20 *μ*m). (b) The bar graphs showed the statistical results of apoptotic cells accounts. The positive cell levels were counted in 10 random fields of relevant region (40x magnification). Data were presented as mean ± SD in three separate experiments (*n* = 6; ^*∗*^*p* < 0.05, compared with control group; ^#^*p* < 0.05, compared with DAI 3 d group). Apoptotic index (AI), defined as the number of TUNEL positive cells per mm^2^.

To obtain the role of TLR2 in apoptotic response, TUNEL labeling was performed to assess the level of apoptosis in the cortex of rats. TUNEL-positive cells were rarely found in control group. Total number of apoptotic cells was consistently increased in the DAI 3 d group. Significantly fewer TUNEL-positive cells were found in DAI 3 d + TLR2 siRNA group than in DAI 3 d group. Furthermore, cortex of rats in DAI 3 d + Pam3CSK4 group had significantly more TUNEL-positive cells than in DAI 3 d group (Figure 8).

### 3.7. Role of TLR2 in Glial Response after DAI

**Figure 9:**
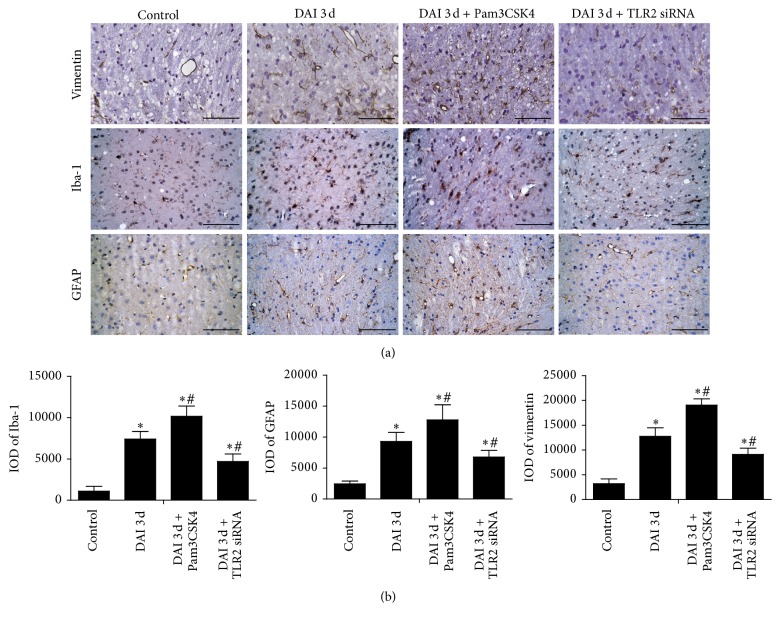
Inhibition or activation of TLR2 was associated with glial reaction. (a) Marker of astrocyte vimentin, GFAP, and marker of microglial cells Iba-1 were assessed by immunohistochemistry in rat cortex (scale bar = 100 *μ*m). (b) The bar graphs showed the statistical results for vimentin, GFAP, and Iba-1 assessed by immunohistochemistry. The relative increases in vimentin, GFAP, and Iba-1 levels were assessed by densitometry (IOD) and positive cell levels were calculated in 10 random fields (40x magnification). Data were presented as mean ± SD in three separate experiments (*n* = 6; ^*∗*^*p* < 0.05, compared with control group; ^#^*p* < 0.05, compared with DAI 3 d group).

In order to determine the role of TLR2 in glial response after DAI, analyses of vimentin, GFAP, and Iba-1 immunostaining were performed in the rat cortex. Cortex in control group displayed vimentin, GFAP, and Iba-1 immunoreactivity with a minimal extent. Staining of star-shaped vimentin positive cells, astrocytes, and microglial cells was more robust and the numbers of vimentin-positive cells, Iba-1-positive microglia, and GFAP-positive astrocytes in the cortex were significantly higher than in the DAI 3 d group. In comparison with DAI 3 d group, significantly fewer and less robust stained vimentin-positive cells, Iba-1-positive microglia, and GFAP-positive astrocytes were found in DAI 3 d + TLR2 siRNA group. There were higher levels of vimentin-positive cells, Iba-1-positive microglia, and GFAP-positive astrocytes in DAI 3 d + Pam3CSK4 group than in DAI 3 d group, and the cells were more extensive, dendritic, and deeply stained (Figure 9).

### 3.8. The Specific Mechanism of TLR2 Downstream Signaling Molecules

**Figure 10:**
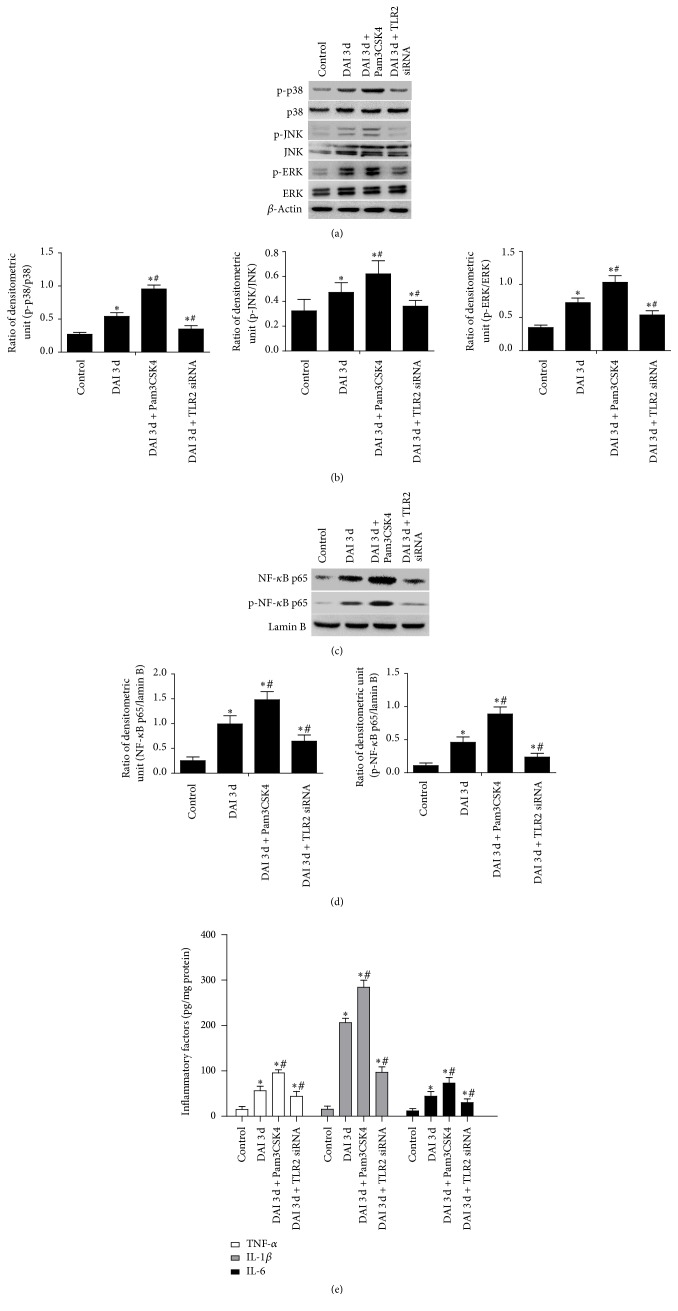
TLR2 signaling pathway induced-inflammatory factors were involved in secondary injury after DAI. (a) Phosphorylation of signaling molecules including p38 MAPK, ERK1/2, and JNK in cortex was examined by western blotting. The expression of *β*-actin was used as an internal control. (b) The bar graphs showed the statistical results for phosphorylation levels of p38 MAPK, ERK1/2, and JNK. (c) Expression of NF-*κ*B p65 and phospho-NF-*κ*B p65 in nuclear extraction of cortex was examined by western blotting. The expression of lamin B was used as an internal control. (d) The bar graphs showed the statistical results for NF-*κ*B p65 and phospho-NF-*κ*B p65 in nuclear extraction. (e) The effects of TLR2 siRNA and Pam3CSK4 on the levels of inflammatory factors including TNF-*α*, IL-1*β*, and IL-6 in rat cortex after DAI were determined by ELISA. All data were presented as mean ± SD in three separate experiments (*n* = 6; ^*∗*^*p* < 0.05, compared with control group; ^#^*p* < 0.05, compared with DAI 3 d group).

Western blotting analysis was performed to examine the expression of signaling molecules of TLR2 pathway after DAI, including TLR2, NF-*κ*B, phospho-NF-*κ*B, p38 MAPK, phospho-p38 MAPK, ERK1/2, phospho-ERK1/2, JNK, and phospho-JNK. The results showed that TLR2 inhibition by siRNA significantly downregulated the expression of TLR2, NF-*κ*B, phospho-NF-*κ*B, and phosphorylation of p38 MAPK, ERK1/2, and JNK in rat cortex compared with DAI 3 d group, while TLR2 agonist, Pam3CSK4, has opposite effects (Figures 10(a)–10(d)). Furthermore, the levels of inflammatory factors including TNF-*α*, IL-1*β*, and IL-6 in rat cortex were determined by ELISA. The results showed that the levels of these inflammation factors were significantly increased in DAI 3 d group compared with control group but were decreased in DAI 3 d + TLR2 siRNA group. Besides, levels of inflammatory factors in DAI 3 d + Pam3CSK4 group were significantly higher than in DAI 3 d group (Figure 10(e)).

## 4. Discussion

The underlying relationship between TLR2 induced inflammation and secondary injury after DAI is yet to be completely demonstrated. In this study, we discovered that TLR2 activation by Pam3CSK4 exacerbated cortical apoptosis, glial response, and neuronal and axonal damage in a rat DAI model, by elevating levels of the proinflammatory cytokine, IL-6, IL-1*β*, and TNF-*α* via NF-*κ*B and MAPK pathway. Furthermore, transient transfection of DAI rat brain with TLR2 siRNA could play a neuroprotective role by significantly abrogating DAI-induced TNF-*α*, IL-1*β*, and IL-6 secretions.

DAI, caused by acceleration and deceleration induced-strain and tearing of the axonal fibers, is a result of the sudden movement of the head. Areas commonly affected include axons in the brainstem, parasagittal white matter near the cerebral cortex, corpus callosum, and internal capsule. In this study, the DAI model was established by an instant rotational acceleration device. The pathological process of DAI was simulated by this model. To identify the specific pathological changes associated with axonal injury, morphological observations were performed in the frontal cortex. H&E staining showed the pathological features of acute neuron injury, such as neuronal pyknosis, swelling, torsion, and cell body deformation. Silver staining exhibited bead-like structures and axonal retraction balls. In addition, increased *β*-APP and NF-L, decreased tau, and impaired axon assessed by TEM, all could be observed in the DAI model, which also demonstrated that the DAI model was successfully established.

Previous study demonstrated that TLR2 played different roles in CNS diseases. Applying TLR2 agonists substantially rescued ischemia induced oligodendrocyte death and reduced ischemic demyelination in vivo [12]. TLR2 appeared to be neuroprotective in cerebral ischemia/reperfusion injury, by enhancing the activation of protective signaling pathways [24]. Moreover, TLR2 agonist robustly increased the microglial response to ablation and reduced secondary degeneration of central myelinated fibres following laser-induced spinal cord injury [11]. Wallerian degeneration, axonal regeneration, and recovery of locomotor function were also impaired in TLR2-deficient mice after sciatic nerve lesion [10]. However, TLR2-induced microglial activation propagated stroke-induced CNS injury, exacerbated pain hypersensitivity after spinal cord injury, contributed to kainic acid mediated innate immune responses and hippocampal excitotoxicity, and aggravated intracerebral hemorrhage-induced brain damage [25–28]. Protective treatment measures against transient focal ischemia-induced brain damage, such as ischemic post conditioning, hyperbaric oxygen, DIDS (4,4′-diisothio-cyanostilbene-2,2′-disulfonic acid), and agmatine, were all associated with inhibition of neuroinflammation via suppressing the TLR2 pathway [29–32]. In this study, we presented that inhibition of TLR2 exhibited a neuroprotective effect in the secondary injury through inflammatory agents in DAI and TLR2 activation by Pam3CSK4 exacerbated cortical apoptosis, glial response, and neuronal and axonal damage, through NF-*κ*B and MAPK pathways by elevating levels of the proinflammatory cytokine.

Diffuse axonal injury usually occurs in subcortical, central, and brainstem white matter tracts [33]. In this study, we firstly examined the expression of TLR2 at different time points in the brain. Increased TLR2 immunoreactivity was observed in the cortex, corpus callosum, and internal capsule of DAI animals and peaked at 3 d and sustained at 7 d following DAI, indicating that TLR2 was activated in the injured tissue. Our data also demonstrated that microglial cells mainly expressed TLR2 after DAI, in accordance with previous research that TLR2 were activated in glial cells in response to tissue injury, including trauma, hemorrhagic shock, and ischemia [34–36].

The effect of IL-1*β*, IL-6, and TNF-*α* in secondary neural injury following trauma has been fully confirmed. An increase in *β*-APP was obvious in white matter axonal tracts and the overexpression of IL-1*β* was strongly related to axonal injury, while IL-6 mRNA and protein expression were also positive at sites where axonal injury was observed [37, 38]. Besides, TNF-*α* could directly induce primary demyelination and oligodendrocyte apoptosis [39]. In this study, we used Pam3CSK4, a TLR2 agonist, to boost TLR2 expression in vivo. After Pam3CSK4 stimulation, expression of TLR2 and NF-*κ*B and phosphorylation of NF-*κ*B, p38 MAPK, ERK1/2, and JNK were significantly increased in cerebral cortex as well as TNF-*α*, IL-1*β*, and IL-6. However, such effects could be reversed by inhibition of TLR2 with TLR2 siRNA transfection. Moreover, we also demonstrated that increased expression of IL-1*β*, IL-6, and TNF-*α* was associated with *β*-APP expression, cell apoptosis, and total tau level. Previous studies also showed that the secretion of IL-6, IL-1*β*, and TNF-*α* was associated with TLR2-activated MAPK (ERK1/2, JNK, p38 MAPK) and NF-*κ*B signaling pathways [40–45]. Collectively, it is reasonable to hypothesize that DAI-induced secondary injury might be depending on cytokine secretions and via TLR-2/NF-*κ*B and MAPK signaling cascade.

So far, the direct mechanism of glial cell activation in DAI is not entirely clear. TLR2 deficiency affected microglial proliferative expansion in response to stereotactic transection of axons, which identify a role for TLR2 signaling in the early glial response to brain injury, acting as an innate bridge to neuroinflammation [46]. It has also been reported that necrotic neuronal cells induce glial cell activation via TLR2 in vitro and in vivo after TBI [46, 47]. In this study, we observed that TLR2 agonist or siRNA transfection was associated with glial cell reaction characterized by GFAP and vimentin positive astrocyte and Iba-1 positive microglia, which indicated that endogenous TLR2 agonist released from the necrotic neurons in the injury site may induce glial cell activation nearby. Alternatively, it is also likely that glial activation in DAI rats results from the release of TLR2-induced-inflammatory cytokines, such as IL-6, IL-1*β*, and TNF*α*, but future studies are needed to verify this possibility.

In addition, there are certain limitations in our current study, which will be addressed in further studies: (1) investigation of specific types of DAMP molecules involved in TLR2 activation has been either neglected or limited by diagnostic challenges. The specific types of the DAMP molecules released from injured neurons after DAI remain uninvestigated. (2) This study, which was performed on animals, held significant potential but was a long way from being offered to patients. Human epidemiological and pathophysiological studies attempting to link TLR2 activation and axonal damage induced by trauma should henceforth put more emphasis on the underlying mechanisms.

## 5. Conclusion

This study suggested that TLR2 activation by Pam3CSK4 exacerbated cortical cell apoptosis, glial response, and neuronal and axonal damage, through NF-*κ*B and MAPK pathway by elevating levels of the proinflammatory cytokines, IL-6, IL-1*β*, and TNF-*α* in a rat DAI model. Although further research is required to fully elucidate the role of TLR2 pathway in the DAI brain, the present study demonstrated, for the first time, TLR2 pathway may play an important role in secondary injury and the inhibition of TLR2 by siRNA could provide neuroprotection in secondary injury after DAI.
